# Peri-ictal Water Drinking in an Omani Patient with Bilateral Mesial Temporal Sclerosis

**DOI:** 10.18295/squmj.12.2023.089

**Published:** 2024-05-27

**Authors:** Younis Al-Mufargi, Haifa Alabri, Arunodaya Gujjar

**Affiliations:** 1 Department of Human Clinical Anatomy, Sultan Qaboos University, Muscat, Oman; 3 Department of Medicine, Sultan Qaboos University, Muscat, Oman; 2 Department of Medicine, Sultan Qaboos University Hospital, Sultan Qaboos University, Muscat, Oman

**Keywords:** Peri-Ictal Water Drinking, Ictal Spitting, Epilepsy, Autonomic

## Abstract

Peri-ictal water drinking (PIWD) is a rare vegetative manifestation of temporal lobe epilepsy without a definite lateralisation value. We report a case of PIWD in a 22-year-old Omani male patient with post-concussion syndrome and epilepsy presented to a tertiary care hospital in Muscat, Oman, in 2021 for evaluation of paroxysmal events. His behaviour of PIWD was misinterpreted by his family until characterised in the epilepsy-monitoring unit as a manifestation of epilepsy that was treated medically. To the best of the authors’ knowledge, this is the second reported case in the region.

Vegetative manifestations have been well-described in patients with epilepsy during the seizure in both paediatric and adult age groups.^1^^,^^2^ Peri-ictal vegetative symptoms (PIVS) may occur without any particular relationship to the cause of epilepsy.^3^ PIVS include cardiovascular, respiratory, gastrointestinal and urinary signs and symptoms. Peri-ictal water drinking (PIWD) is an infrequently described automatism in an epileptic patient, often among those with temporal lobe epilepsy.^3^^,^^4^ Among patients with focal epileptic seizure, 65 cases only are reported to have PIWD behavior.^4^ PIWD has been defined as the urge to drink water ictally or up to 2-minute in the post-ictal phase of epileptic seizures.^1^ Peri-ictal water drinking as a manifestation of epileptic seizure signifies a reliable sign of lateralisation to a non-dominant temporal lobe.^5^

In this report, we describe a case of PIWD in a patient with post-concussion syndrome with bilateral mesial temporal sclerosis. To the best of the author’s knowledge, this is the second reported case in the region. It was found that PIWD has localising but no lateralising value.

## Case Report

A 22-year-old right-handed male patient had a road traffic accident at the age of 11 years and suffered a post-concussion syndrome with behavioural changes of impulsiveness and seizures. His seizures were described as chest discomfort followed by lip and hand automatism and excessive water drinking during the event. His family thought that he would drink water to relive his chest discomfort. After that, he was routinely offered a bottle of water whenever his seizure started, in the context of this recurrent habitual behaviour.

**Figure 1:**
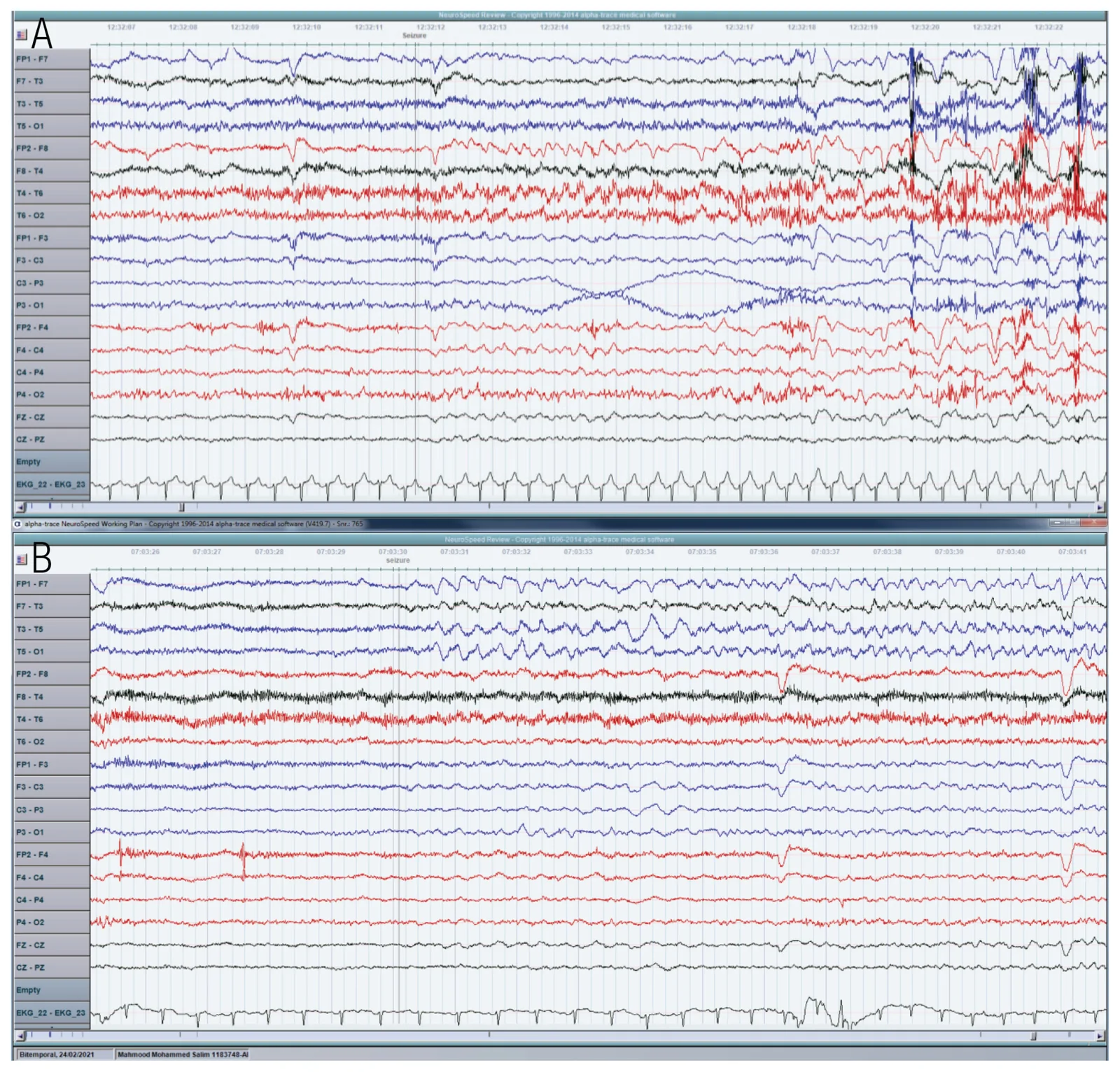
Ictal electroencephalogram onset with rhythmic discharges in the **(A)** right temporal chain, maximum at F8/T4 and (**B)** in the left temporal chain, maximum at T3.

To better characterise his seizure semiology and localise the ictal onset, he was admitted for 3 days in the Epilepsy Monitoring Unit at a tertiary care hospital in Muscat, Oman, for long-term electroencephalogram (EEG) evaluation [Figure 1]. During evaluation, his anti-seizure medications (ASM) including lacosamide 200 mg twice daily, sodium valproate SR 750 mg AM/1000 mg PM and clobazam 15 mg AM/20 mg PM were tapered off. His interictal recording showed intermittent slowing in the right temporal chain and bitemporal sharp waves: 80% on the right (maximum at F8>T4), 20% on the left (maximum at F7>T3). He had 5 seizures of the same semiology. Seizures started with him holding his chest, either due to discomfort or as a reaction to a gastric aura. This was followed by swallowing movements and hand automatism. Out of 5 seizures, 3 seizures had clear water drinking during the seizure or after the seizure terminated. He would ask for or grab a nearby water bottle to drink multiple times. The water bottles were 500 mL, and he drank almost 2/3 of the bottle in each seizure. During the other 2 seizures, the patient was alone and his behaviour seemed restless, looking for water through his bed sheets but not found. He would always ask for water and no other types of fluids.

In seizure 1, he used his hands to gesture to his attendant that he needs to drink. He repeatedly grabbed the bottle from his attendant to drink a second and third time after giving it back from drinking the first time. In the third time, he pointed to his chest as of explaining the reason for drinking [Table 1]. All 3 events of water drinking in seizure 1 happened ictally. In seizure 2, he drank twice ictally and 24 seconds post ictally. In both events, he grabbed the water bottle off his attendant’s hand to drink. In seizure 3, the seizure woke him up from sleep while the attendant was not aware of the event. There were no water bottles close to him, so he seemed restless and looking around the room frequently. Finally, he got off bed and walked around the bed but that was not visualised by the video. In the 4th seizure, again he was alone and seemed restless when the seizure started as the attendant was not around. There was a bottle close to his pillow, but he did not see it. He seemed in discomfort and wiping his face and left nostril multiple times with his hands. In seizure 5, it started again by chest sensation; he held his hands against his chest then started swallowing movements. His attendant walked into the room and the patient pointed by hand gestures that he wants to drink and again pointed to his chest as of explaining his reasoning. He started spitting and he drank all the bottles given to him (~400 mL).

**Figure 2:**
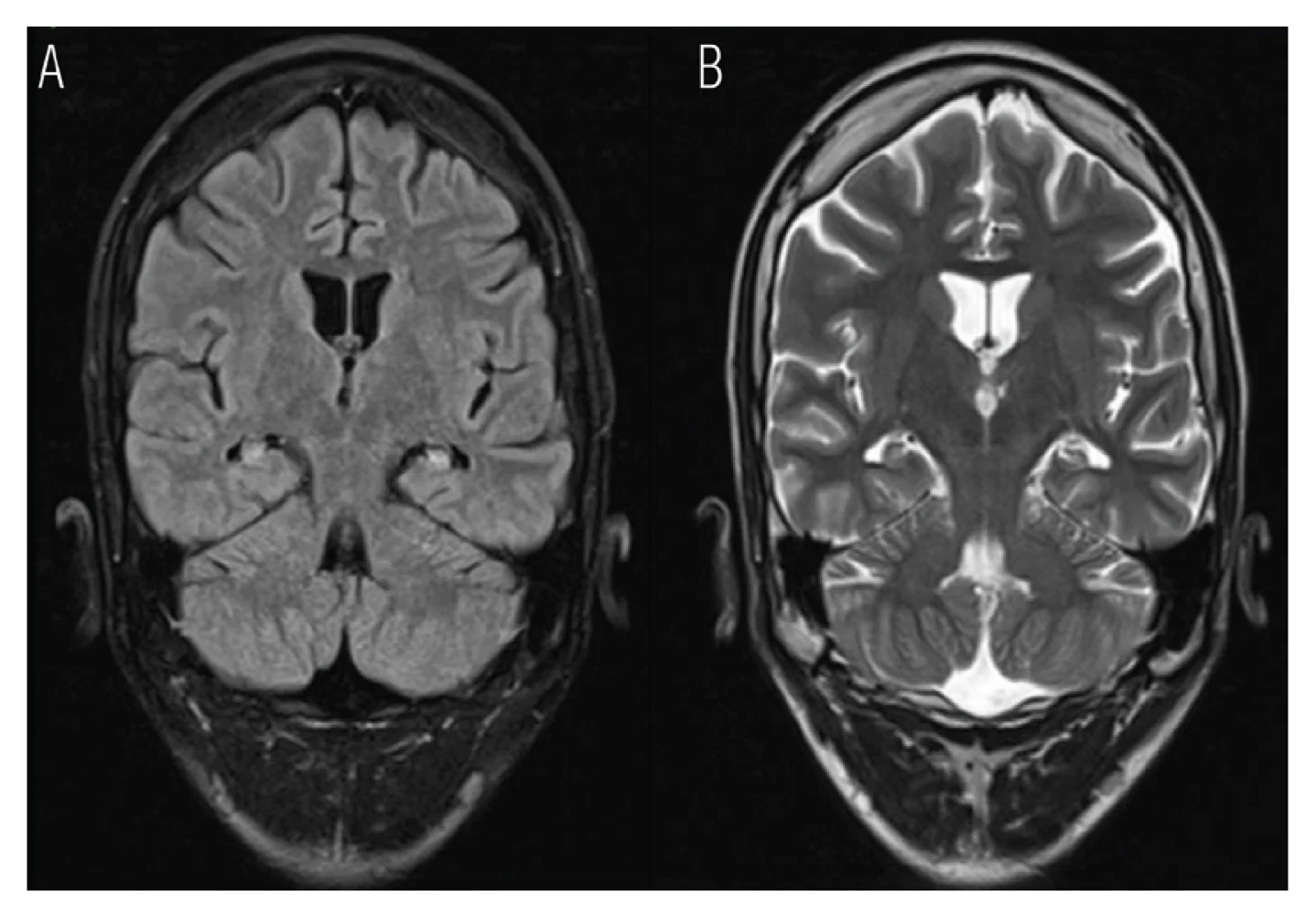
Magnetic resonance imaging of the brain in **(A)** flair and **(B)** T2 that both showed evidence of bilateral hippocampal atrophy and mesial temporal sclerosis.

His magnetic resonance imaging of the brain showed evidence of bilateral hippocampal atrophy and bilateral mesial temporal sclerosis [Figure 2]. His positron emission tomography-computed tomography of brain was unremarkable with no focal hyper or hypometabolism. The patient’s consent was obtained for publication purposes.

## Discussion

Peri-ictal water drinking was first reported upon a statistical review of aura in epilepsy among 1,359 cases by Lennox, defined as the urge of drinking water ictally or up to 2-minutes in the post-ictal phase of epileptic seizures.^1^^,^^6^ Seeking water in epileptic patients has been noticed mostly in ictal phase, yet post-ictal water drinking has been reported as well.^1^ Reported cases of PIWD in literature remain infrequent all over the world, with around 65 cases have been reported to date, including one case in the Middle Eastern region.^4^^,^^7^

Water-seeking behaviour has been correlated electro-clinically and neuropathologically with pathologies in the mesial temporal lobe, emphasising further on the value of localisation hypothesis.^8^ However, a recently published retrospective study in surgically confirmed patients with focal epilepsy in which PIWD occurs more often in frontal lobe epilepsy than temporal lobe epilepsy (TLE) indicating that PIWD might not be a specific symptom of TLE.^9^ The epileptic discharges in the current patient’s seizure confirmed the localisation hypothesis of temporal lobe origin. The epileptic discharges from the temporal lobe structures propagating to the hypothalamus is proposed to be a stimulus for water-seeking behaviour and sense of thirst in epilepsy.^8^^,^^10^

Several cases of PIWD have been published in the literature highlighting the value of lateralisation of peri-ictal water drinking in patients with temporal lobe epilepsy. Water seeking behaviour has been concluded as a sign representing epileptic focus on the non-dominant temporal lobe.^11^^,^^12^ This lateralisation may be explained by asymmetrically represented network of central autonomic system implemented in water-seeking behaviour, control of fluid and thirst.^11^ However, other studies have shown no consistent lateralisation of epileptiform activity in similar cases.^3^^,^^5^^,^^13^ In the current patient, we could not conclude definite lateralisation as he had seizures with water drinking behaviour evident in either of the temporal lobes. Even though, seizures number 1 and 2 that originated from the right temporal lobe started earlier at 12 and 15 seconds compared to seizure number 5 that may argue that it lateralised to the non-dominant hemisphere. However, we have incomplete data of seizures 3 and 4 that may change the equation; therefore, the significance of lateralisation in patients presenting with PIWD is still uncertain as demonstrated in the current case.

The autonomic manifestations of epilepsy have a risk to progress into status epilepticus as reported in the literature.^14^ Therefore, the importance of detecting other signs of epileptic events as autonomic manifestations such as cardiac and respiratory, is very crucial as it may lead to sudden unexpected death in epilepsy.

As noticed in the current case, the patient had also other epileptic signs of temporal origin such as post-ictal spitting. Spitting as an ictal phenomenon which has been evoked by direct electrical stimulation to temporal lobe in many trials, confirming the value of localisation again with no lateralisation value.^15^

Focal epileptic syndromes are estimated to affect 60% of patients with epilepsy, in which 15% of those patients’ condition cannot be controlled by anti-seizure medications adequately and as observed assumption, half of them may be considered potential candidates for a surgical intervention of epilepsy. A published case of middle-aged man diagnosed with epilepsy since childhood with features of PIWD on anti-seizure medications of right temporal lobe origin, remained seizure-free in a 1-year follow-up after right anterior temporal resection.^7^ Moreover, a recent retrospective study discussed the occurrence of PIWD in focal epilepsy patients with favourable outcomes postoperatively, including frontal lobe epilepsy and temporal lobe epilepsy signifying the value of preoperative evaluation in patients with focal epileptic syndromes.^9^ In the present case, the patient had bi-temporal lobe epilepsy secondary to bilateral mesial temporal sclerosis that was better controlled with anti-seizure medications but had significant behavioural issues requiring follow-up with a psychiatrist. A multidisciplinary discussion to consider if he would benefit from surgical management aimed at improvement in both his seizures and behavioural issues is planned.

## Conclusion

PIWD and ictal spitting are infrequent vegetative symptoms seen in the temporal lobe epilepsy without a definite lateralisation value.

## Figures and Tables

**Table 1:** Summary of seizures, electroencephalogram onset, duration and details of water drinking phenomenon in a 22-year-old male patient

	EEG Onset	Seizure duration	Onset of first drinking during a seizure	Number of times drinking during the seizure	Further remarks
Seizure #1	Right temporal	44 sec	12 sec	3 times	
Seizure #2	Right temporal	66 sec	15 sec	2 times during a seizure and 1 after the seizure ends (1.5 min after seizure ended)	
Seizure #3	Left temporal	47 sec	NA	NA	Nocturnal seizure
Seizure #4	Left temporal	54 sec	NA	NA	Left nose wiping
Seizure #5	Left temporal	63 sec	55 sec	1 time	Post Ictal spitting

EEG = electroencephalogram; NA = not available.
